# An Approach for Identifying Brainstem Dopaminergic Pathways Using Resting State Functional MRI

**DOI:** 10.1371/journal.pone.0087109

**Published:** 2014-01-31

**Authors:** Jason Vytlacil, Andrew Kayser, Asako Miyakawa, Mark D’Esposito

**Affiliations:** 1 Helen Wills Neuroscience Institute, University of California at Berkeley, Berkeley, California, United States of America; 2 Department of Psychology, University of California at Berkeley, Berkeley, California, United States of America; 3 Ernest Gallo Clinic & Research Center, University of California at San Francisco, San Francisco, California, United States of America; Beijing Normal University, Beijing, China

## Abstract

Here, we present an approach for identifying brainstem dopaminergic pathways using resting state functional MRI. In a group of healthy individuals, we searched for significant functional connectivity between dopamine-rich midbrain areas (substantia nigra; ventral tegmental area) and a striatal region (caudate) that was modulated by both a pharmacological challenge (the administration of the dopaminergic agonist bromocriptine) and a dopamine-sensitive cognitive trait (an individual’s working memory capacity). A significant inverted-U shaped connectivity pattern was found in a subset of midbrain-striatal connections, demonstrating that resting state fMRI data is sufficiently powerful to identify brainstem neuromodulatory brain networks.

## Introduction

Dopamine is critical for a myriad of different functions ranging from motor control to high-level cognition [1], and is implicated in the pathology of neurological and psychiatric disorders such as Parkinson’s disease and schizophrenia. The midbrain, particularly the substantia nigra and ventral tegmental area, is the site of the richest concentration of dopaminergic neurons in the brain, which project densely to the striatum but also to others regions, most notably the prefrontal cortex and hippocampus [2]. Here, we present an approach for identifying brainstem dopaminergic pathways using resting state functional MRI in humans.

Numerous studies using resting state fMRI data have shown that neuronal activity is characterized by temporal correlations in blood oxygen level-dependent (BOLD) signal across disparate brain regions [3]. These fluctuations seem highly consistent over time and reflect the presence of intrinsic functional [4] and structural [5] connectivity. To date, brainstem neuromodulatory projections, such as those comprising the nigrostriatal dopaminergic system, have not been identified in resting state fMRI data. Thus, in a group of healthy individuals, we searched for significant functional connectivity between dopamine-rich midbrain areas (substantia nigra; ventral tegmental area) and a striatal region (caudate) that was modulated by both a pharmacological challenge (the administration of the dopaminergic agonist bromocriptine) and a dopamine-sensitive cognitive trait (an individual’s working memory capacity). Given the strong evidence that the relationship between cognitive performance and dopamine levels follows an inverted-U shaped function [6], and that working memory capacity can be linked to dopamine synthesis capacity in the caudate [7], we reasoned that voxels of the midbrain and caudate that varied in this manner would likely reflect dopaminergic pathways.

## Materials and Methods

### Ethics Statement

All procedures were approved by The University of California at Berkeley Committee for the Protection of Human Subjects. All participants gave their written informed consent.

### Participants

Eighteen healthy subjects (ages 18–22, 8 males) underwent two fMRI sessions after receiving a dopaminergic agonist (1.25 mg bromocriptine), or placebo, in a randomized, counterbalanced, double-blind design. Two subjects were excluded after realignment parameters indicated rapid head movement in a direction larger than the voxel size in that dimension.

### Cognitive Task

Baseline working memory capacity was measured by performance on the listening version [8] of the Daneman and Carpenter reading span task [9]. In this task, subjects listened to a series of sentences for which they were required to remember the last word of each sentence in the order of presentation. The number of sentences increased across trials, and the span score was based upon the number of words correctly recalled. Subjects were divided into low- and high-span groups based on the span task score via a median split, with individuals attaining a score of 3.5 and above considered high-span. As a result, 8 subjects were classified as high-span, and 8 subjects as low-span.

### MRI Data Acquisition and Analysis

MRI data for both the bromocriptine and placebo sessions were acquired on separate days from the behavioral data using a Siemens 3T Trio Magnetom scanner with a 12-channel head coil. Five minutes of functional data were collected (gradient echo-planar pulse sequence; TR = 2 s, TE = 32 ms, flip angle = 90; 27 oblique slices, voxel size 1.8×1.8×3.45 mm) from subjects while they rested with their eyes open. Echo planar images (EPI) were slice-time corrected and realigned via SPM 5 tools. EPI volumes were then co-registered to each subject’s T1-weighted image, band pass filtered (0.009<f<0.08) to remove fluctuations outside the dynamic range of the hemodynamic response function, and spatially smoothed (gaussian kernel - FWHM 4 mm). A mean time series was extracted from nuisance ROIs (white matter and ventricles, CSF segmented from T1 images via FSL tools [10]) and regressed out of EPI data.

**Figure 1:**
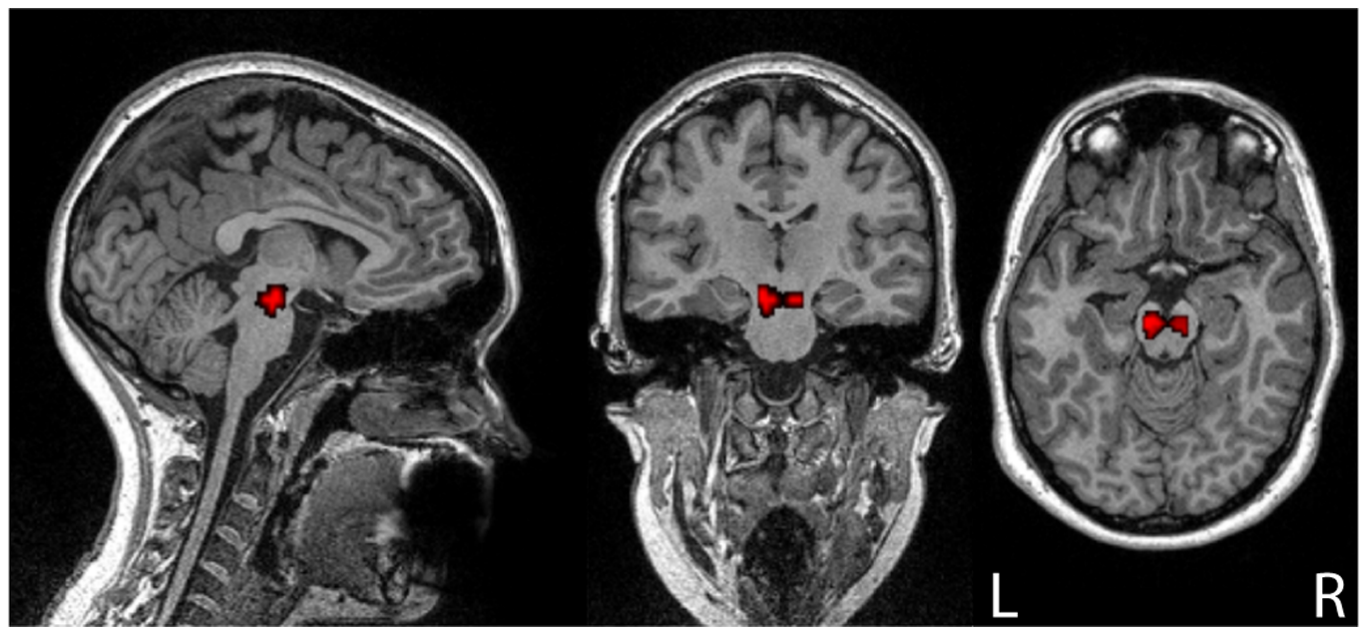
Representative bilateral midbrain ROI (red) on a co-registered T1 image in one participant.

High-resolution T1-weighted images were collected using a MP-RAGE sequence (TR = 2.3 s, TE = 2.98 ms, voxel size: 1×1×1 mm) to enable identification of the substantia nigra and the ventral tegmental area (VTA). ROIs comprising these dopaminergic midbrain nuclei were created on each subject’s T1 image via visual inspection and a fixed set of fill parameters using the 3dfill tool within mricron (http://www.cabiatl.com/mricro/mricron/stats.html) to ensure regularity of the ROI across subjects. **Figure 1** shows a representative midbrain ROI on a coregistered T1 image in one participant. The mean time series from midbrain ROIs was extracted and Pearson's correlation coefficient was calculated with all other voxel time series throughout the brain, creating a connectivity map. R values were transformed to z-scores via the Fisher transformation (i.e. hyperbolic arctangent) [11]. Co-registered T1 images were normalized to the Montreal Neurological Institute Atlas via SPM 5 tools. Normalization parameters were used to normalize correlation maps from all subjects to a common atlas space. Bilateral caudate, putamen and ventral striatum ROIs were defined using the Talairach Daemon atlas with WFU Pickatlas software [12]–[13].

## Results

**Figure 2:**
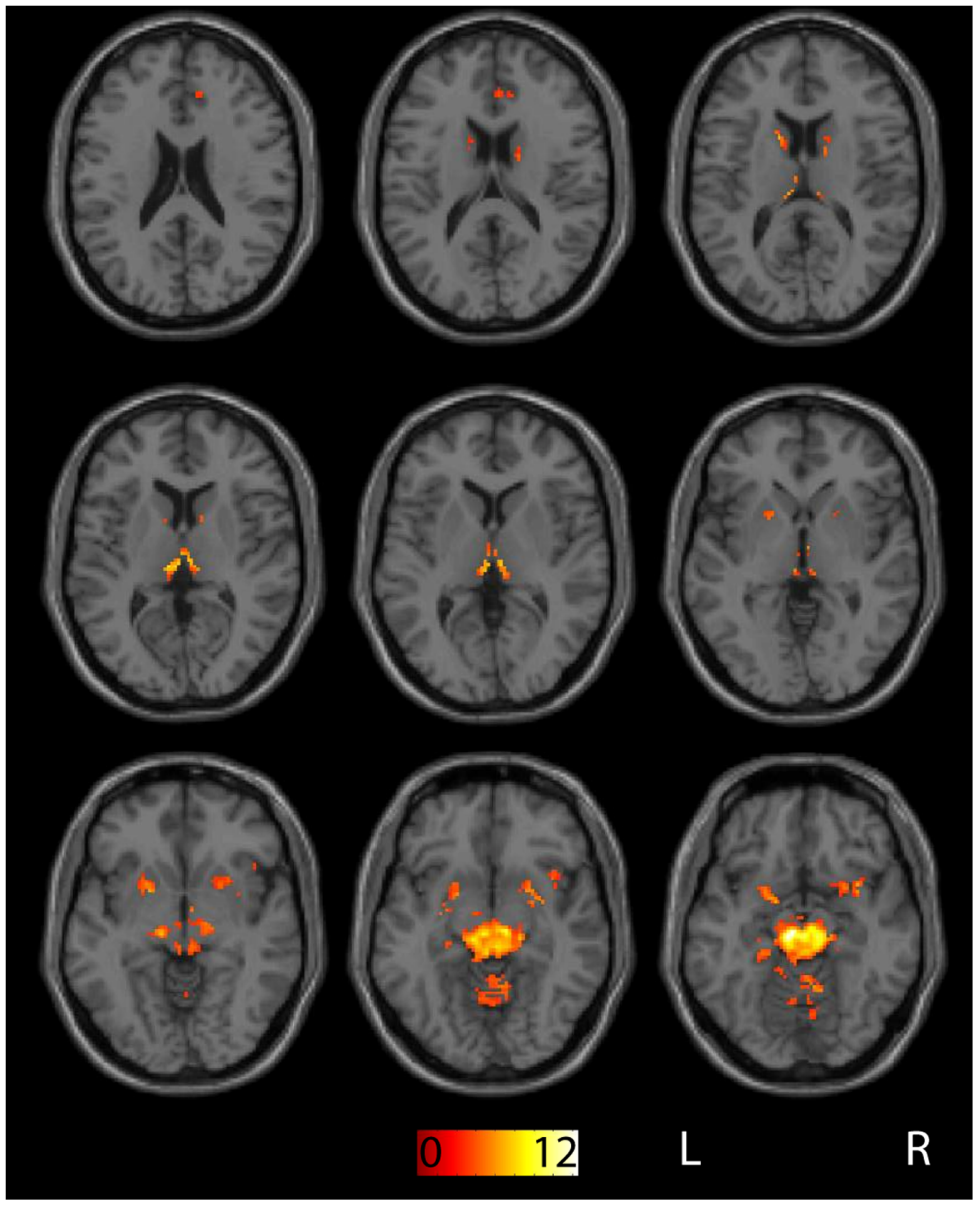
Whole-brain correlation maps with midbrain seeds across all subjects and across both placebo and bromocriptine sessions. Maps are thresholded at p < .05 FDR corrected with a minimum cluster size of 20 voxels. The color bar indicates t values.

Correlation maps with midbrain seeds were created and the results were analyzed for group level effects. A t-test was performed across maps from all subjects, including both placebo and drug sessions. Significant functional connectivity with the midbrain, irrespective of drug condition, was observed in the bilateral thalamus, dorsal and ventral striatum, globus pallidus, medial frontal cortex, medial temporal lobes and cerebellum (see **Figure 2**).

We sought to further investigate whether the observed midbrain connectivity with other brain regions actually reflected dopaminergic projections by assessing whether connectivity was modulated by both the administration of the dopaminergic agonist bromocriptine and an individual’s working memory capacity (a dopamine sensitive cognitive trait). Prior human studies have demonstrated that the effects of dopaminergic drugs on cognition depend on an individual’s baseline working memory capacity, as measured by span tasks [14]. Specifically, dopaminergic agonists improve cognition in low-span subjects, but impair cognition in high-span subjects, consistent with an inverted-U shaped model of dopamine function [6]. This finding likely reflects different baseline dopamine levels among individuals (low span - low dopamine; high span - high dopamine). With PET scanning, we previously confirmed this hypothesis by finding a significant positive correlation between dopamine synthesis capacity in the left caudate nucleus and working memory span [7]. Thus, if midbrain-caudate connectivity reflects dopaminergic pathways, the magnitude of this relationship in resting data should exhibit an inverted-U shaped function, based on an individual’s working memory capacity (as measured by the listening span test [8]) and the drug administered.

**Figure 3:**
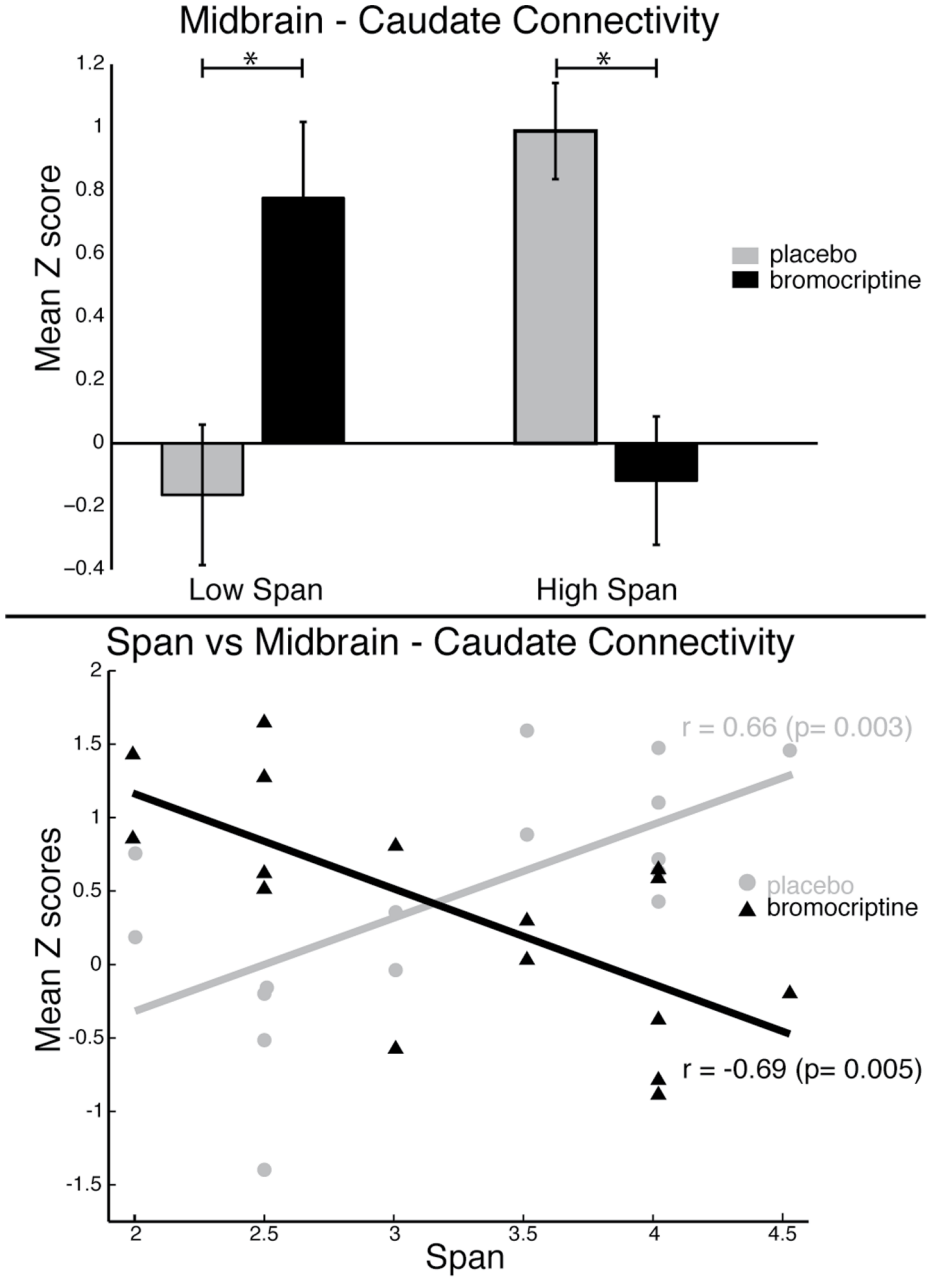
Correlations between midbrain and left caudate activity: voxels demonstrating a significant inverted-U shaped effect. ** Top.** Mean Z scores of correlations of midbrain-caudate connectivity divided into span groups and drug conditions. Error bars indicate standard error. **Bottom.** Mean Z scores of correlations of midbrain-caudate connectivity with performance on the listening span test during placebo or drug condition. Working memory span was measured as the number of words recalled on the listening span task (see Methods).

Midbrain correlation values were extracted from the bilateral caudate voxels in all subjects and sessions and entered into a 2×2 ANOVA with factors of drug (bromocriptine vs placebo) and span (high vs low). A quadratic contrast (−0.5 0.5 0.5 −0.5) was then created to determine whether these voxels were consistent with a hypothesized inverted-U shaped function. A significant interaction between drug and span was found in voxels within the left caudate nucleus (p < .05, FDR, small volume correction). These caudate voxels exhibited increased connectivity with the midbrain in low span subjects on bromocriptine relative to placebo, whereas the opposite effect was found in high span subjects (see **Figure 3**). On the top of **Figure 3**, midbrain-caudate connectivity values from these voxels are plotted separated by span group and drug condition, illustrating the inverted-U shaped response. On the bottom of **Figure 3**, midbrain-caudate connectivity values are plotted against span scores across subjects in both the placebo and drug conditions. Consistent with an inverted-U shaped function, a significant *positive* correlation between midbrain-caudate connectivity and span was found in the placebo condition (r = 0.66, p = 0.003). That is, individuals with higher span had higher midbrain-caudate connectivity. In the drug session, there was a significant *negative* correlation between midbrain-caudate connectivity and span (r = −0.69, p = 0.005). Notably, this correlation was significantly greater in the placebo than the drug condition (Z = 4.14, p < 0.0001). Removing the outlier low span subject did not affect the significant group difference shown in **Figure 3**.

**Figure 4:**
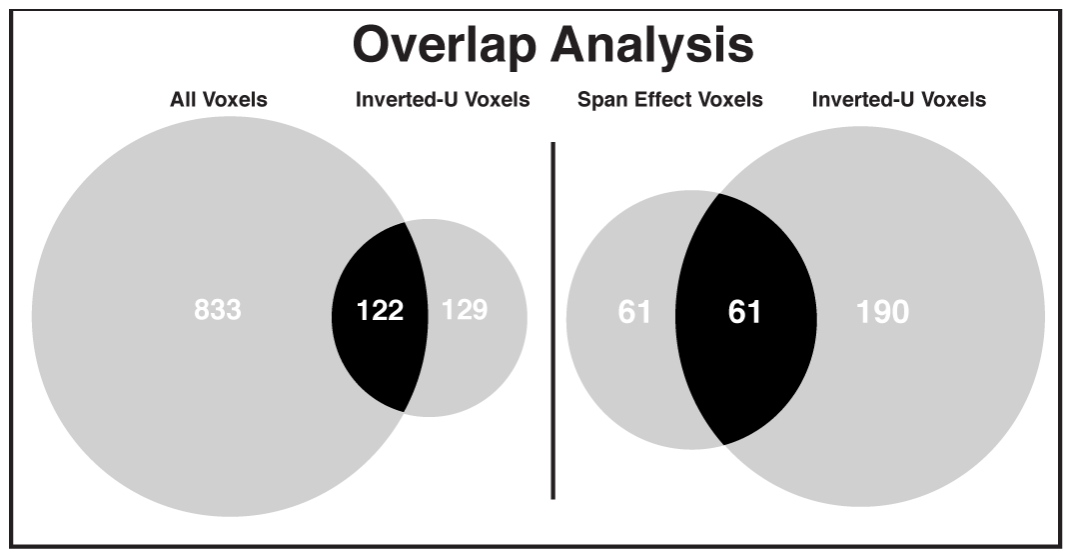
Venn diagrams illustrating degree of overlap. **Left.** Venn diagram illustrates the degree of overlap between caudate voxels identified as correlated with the midbrain (p < 0.05, FDR and small-volume corrected) collapsed across span and drug condition (left circle) and caudate voxels identified as having an inverted-U shaped relationship with the midbrain (p < 0.05, uncorrected; right circle). Only 122/(122+833) voxels (13%) exhibiting significant midbrain-caudate connectivity also exhibit an inverted –U shaped response dependent on span and drug. **Right.** Venn diagram illustrates the degree of overlap between caudate voxels identified as having greater connectivity with the midbrain in higher span subjects in the placebo sessions (left circle) and caudate voxels identified as having an inverted-U shaped relationship with the midbrain (p < 0.05, uncorrected). Only 61/(61+61) voxels (50%) exhibiting a significant increase in midbrain-caudate connectivity based on span also exhibit an inverted-U shaped response dependent on span and drug. The number of voxels is presented within the circles.

We also sought to determine whether caudate voxels that exhibited an inverted-U shaped connectivity pattern with the midbrain across subjects were the same caudate voxels that exhibited significant correlations with the midbrain across all subjects and drug conditions. In other words, could these caudate voxels be identified solely on the strength of their connectivity with presumptive dopaminergic regions in the midbrain, independent of span and response to bromocriptine? Our motivation for this analysis was to determine whether there is added value in using a proxy for measuring brain dopamine (e.g. the combination of span and dopaminergic agonist administration) for identifying dopaminergic pathways in resting fMRI data. These results, presented on the left side of **Figure 4** as a Venn diagram, illustrate that there is only a small degree of overlap between caudate voxels (either left- or right-sided) exhibiting inverted-U shaped connectivity and those identified by correlation across all subjects and drug sessions. Specifically, even when voxels demonstrating the U-shaped response are loosely thresholded (p < 0.05, uncorrected), it can be seen that only 13% of the voxels (122/955 voxels) that exhibit significant midbrain-caudate connectivity across all subjects and drug sessions (left circle) also show an inverted-U shaped response dependent on span and drug (intersection of circles). This percentage is further reduced when a higher, but still relaxed, statistical threshold is applied (26/743 or 4% overlap at p < 0.01, uncorrected).

It is also conceivable that span alone may reliably identify these voxels. However, if one only considers the caudate voxels that exhibit increased connectivity with midbrain for high span subjects compared to low span (a smaller set of voxels, indicated in the leftmost circle in the right-hand Venn diagram), it can be seen on the right side of **Figure 4** that only 50% of these voxels (61/122 voxels) also show an inverted-U shaped response dependent on both span and drug (intersection of circles, right-hand cell) – again, even when these voxels are loosely thresholded (p < 0.05 uncorrected). This percentage is reduced when a slightly higher statistical threshold is applied (4/28 voxels or 14% overlap at p < 0.01, uncorrected). Thus, these findings indicate that most “dopaminergic pathway” voxels would remain unidentified without considering the effect of a dopaminergic drug and dopamine-dependent behavioral measures (i.e. an individual’s working memory capacity).

**Figure 5:**
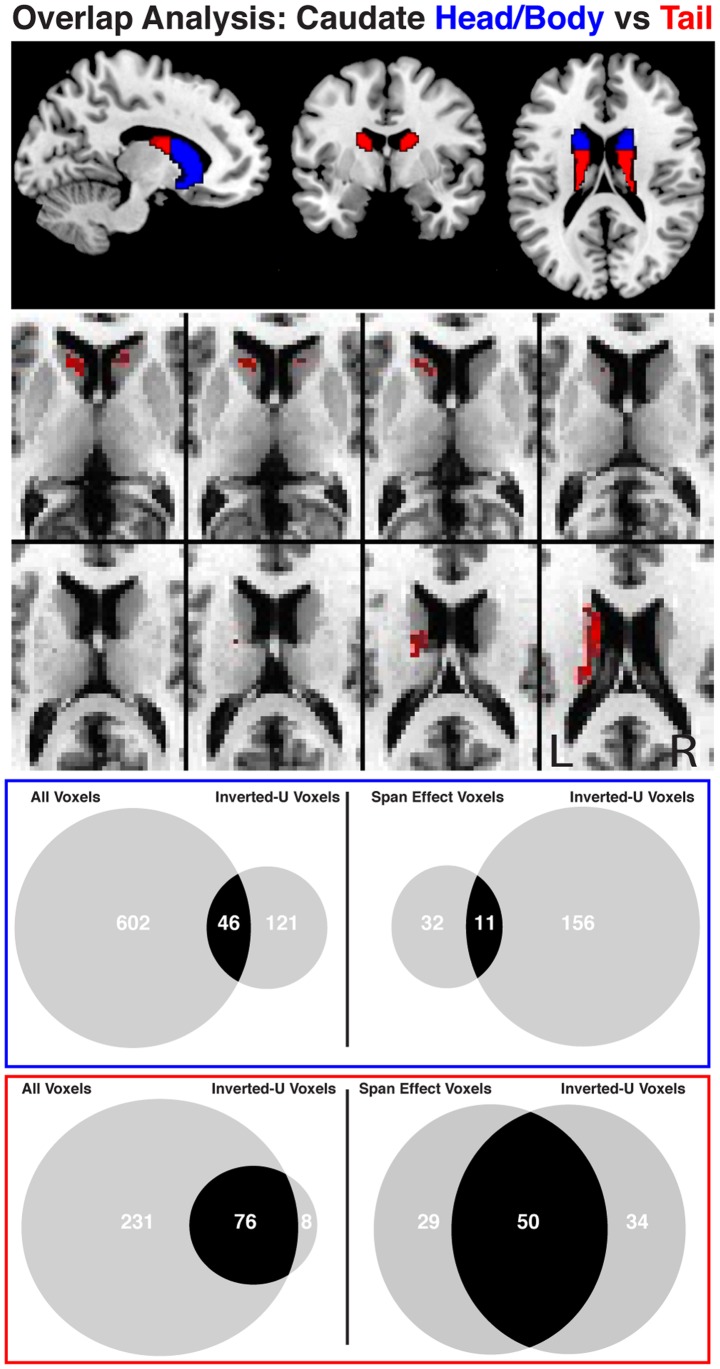
Division of the caudate into a head/body and tail regions is displayed at the top of the figure. Venn diagrams illustrate the degree of overlap between caudate voxels (middle figure – head/body; bottom figure – tail) identified as correlated with the midbrain (p < .05, FDR and small-volume corrected) collapsed across span and drug condition (left circle) and caudate voxels identified as having an inverted-U shaped relationship with the midbrain (p < 0.05, uncorrected; right circle). A significantly greater percentage of overlapping voxels are present in the tail than the head/body of the caudate.

We further sought to investigate whether these potentially dopaminergic voxels were distributed homogeneously throughout the caudate. Loosely-thresholded voxels demonstrating midbrain connectivity profiles consistent with an inverted U-shaped function (p < 0.05, uncorrected) were observed throughout the head, body and tail of the caudate nucleus (see **Figure 5**, top). Evidence from anatomical investigations suggests the existence of multiple distinct circuits in the caudate, each receiving input from its own cortical region [15]. In a recent meta analysis using imaging data from the BrainMap database [16]–[18] Robinson et al [19] were able to segregate activity in the head and body of the caudate, thought to be primarily “cognitive”, from activity in the tail, thought to be primarily “motor”. Thus, we divided our caudate mask into head/body and tail regions by estimating the position of the interventricular foramina of Monro [19]. The head/body was defined to be all caudate voxels anterior to a coronal slice at MNI y = 2 whereas all other caudate voxels were defined to be the tail (see **Figure 5**, middle). This division yielded 1562 and 394 voxels in the head/body and tail respectively. We then performed the same overlap analysis as performed on the entire caudate in each region separately (see **Figure 5** **,** bottom). The proportion of overlap in the caudate tail was significantly higher than that in the head/body (Χ^2^ (1, *N* = 394,1562) = 58.28, *p* = 2.27×10^−14^). This difference was even larger in voxels displaying a span effect (Χ^2^ (1, *N* = 394,1562) = 75.13, *p* = 4.4×10^−18^).

Even though only the caudate exhibited a u-shaped response in our map-wise analysis, we also performed an exploratory search for such effects in other midbrain dopaminergic targets in the basal ganglia. Thus, voxels from the putamen and ventral striatum were subjected to the same overlap analysis described above, quantifying the degree to which they showed connectivity with the midbrain and exhibited changes in connectivity consistent with the inverted-U shaped function. Unlike the caudate, neither of these regions contained voxels that exhibited an inverted-U shaped response dependent on span and drug at an appropriate small volume correction. Even at a relaxed statistical threshold of p < .05 (uncorrected), only 69 of 2073 voxels in the putamen showed an inverted-U response. In the ventral striatum, no voxels showed an inverted-U response, although the small size of this region (47 voxels) may make a null result more likely.

## Discussion

These fMRI data demonstrate that significant correlations of spontaneous BOLD activity exist between the midbrain and the caudate during the resting state. The likelihood that these functionally connected regions reflect dopaminergic pathways is supported by our finding that dopaminergic augmentation with bromocriptine modulated midbrain-caudate connectivity. Moreover, this drug effect exhibited an inverted U-shaped response when an individual’s working memory capacity was also considered. That is, individuals with low baseline working memory capacity (with presumably lower dopamine levels) exhibited increased midbrain-caudate connectivity following administration of bromocriptine, whereas those individuals with high baseline working memory capacity exhibited decreased midbrain-caudate connectivity.

Only a limited number of studies to date have investigated patterns of connectivity within putative dopaminergic circuitry after the administration of selective dopaminergic agonists, and most have done so with data acquired during the performance of a behavioral task. Moreover, all of these studies have only reported changes in frontal-striatal connectivity with dopaminergic modulation [20]–[22] rather than in midbrain-striatal connectivity. One relevant study examined the effect of dopaminergic modulation of frontal-striatal circuitry during the performance of a working memory task [23]. It was observed that frontal-striatal connectivity varied in an inverted-U shaped manner and correlated with performance. Other dopaminergic studies with other cognitive tasks have obtained similar findings. A study by Honey and colleagues found that the selective D2 antagonist sulpiride increased midbrain-caudate connectivity, while the dopamine reuptake inhibitor methylphenidate decreased it [24]. Using structural equation modeling to assess effective connectivity between midbrain, caudate, thalamus, and prefrontal cortex, these authors also noted significant drug-induced changes in the directional path from midbrain to caudate: as above, sulpiride increased the effective connectivity and methylphenidate decreased it. In summary, all of these studies demonstrate that an inverted-U shaped pattern of connectivity can be demonstrated in midbrain-striatal-frontal circuitry.

In contrast with our findings in the caudate, neither putamen nor ventral striatum contained voxels that exhibited an inverted-U shaped response dependent on span and drug when appropriately thresholded, a result that did not change markedly even when these voxels were evaluated at reduced statistical significance. This finding may reflect the fact that dopamine synthesis in the caudate, but not other striatal regions, has been shown to correlate with working memory capacity [7], and is consistent with the particular sensitivity of the caudate to dopaminergic modulation [24]. On the other hand, the lack of significant findings in these regions may indicate the need for more sensitive measurement techniques or the use of other cognitive tasks.

An important point that emerges from our study is that not all significant correlations between midbrain and caudate voxels in resting fMRI data likely represent dopaminergic pathways. In addition, the correlations we observed may not necessarily result solely from correlated activity induced by monosynaptic connections between midbrain and striatum. In keeping with these ideas, our overlap analysis revealed that only a small proportion of voxels exhibiting correlated activity between the midbrain and striatum in resting state BOLD data demonstrated effects consistent with dopaminergic modulation. Further, even within a given structure like the caudate, our analysis reveals regional variation in the proportion of correlated voxels that were likely to be dopaminergic. What may be the source of significant correlations between other midbrain and caudate voxels? One source may be neural in origin. For example, not all VTA projections from the midbrain to the striatum are dopaminergic [25]. Another source may be non-neural in origin. For example, physiological processes such as cardiac pulsatility and respiration [26] can lead to increased correlations between any two brain regions, especially brainstem structures. Notably, these latter processes should not affect the results of our analysis as the drug and span factors interact to produce effects in opposing directions. Future studies should be aimed at identifying these other sources of brainstem-basal ganglia connectivity in BOLD data. In summary, we argue that sensitivity to dopamine manipulations, as we have presented here, represents an important approach for defining and confirming putative dopamine connections arising from the midbrain.

These data also provide constraints on potential neural mechanisms. Bromocriptine increased mesostriatal connectivity in subjects with low baseline dopamine tone, but tended to decrease it in subjects with high baseline dopamine tone. Because D2 receptors can be found both pre- and post-synaptically [27], these findings suggest that the targets of bromocriptine may be influenced by dopamine tone – e.g. that in the context of higher dopamine tone, presynaptic D2 autoreceptors may be more rapidly engaged, thereby reducing dopamine signaling and mesostriatal functional connectivity, than in the case in which dopamine tone is low. While speculative, such models emphasize that work in other animals would benefit from a focus on individual differences in dopamine tone, as well as their impact on circuit function, when considering the mechanisms underlying the effects of dopamine manipulations.

In conclusion, our results suggest that brainstem neuromodulatory systems can be identified in resting state fMRI data, potentially providing an exciting new avenue for investigating the link between brainstem neuromodulatory function and behavior.

## References

[1] Cools R, D’Esposito M (2009) Dopaminergic modulation of flexible control in humans. In: Bjorklund A, Dunnett SB, Iversen LL, Iversen SD, editors. Dopamine Handbook. Oxford: Oxford University Press. pp. 249–261.

[2] Haber S, Fudge J, McFarland N (2000) Striatonigrostriatal pathways in primates form an ascending spiral from the shell to the dorsolateral striatum. J Neurosci 20(6): 2369–2382. 10704511 10.1523/JNEUROSCI.20-06-02369.2000 PMC6772499

[3] Fox M, Snyder A, Vincent J, Corbetta M, Van Essen D, et al (2005) The human brain is intrinsically organized into dynamic, anticorrelated functional networks. Proc Natl Acad Sci U S A 102: 9673. 15976020 10.1073/pnas.0504136102 PMC1157105

[4] Shehzad Z, Kelly AMC, Reiss PT, Gee DG, Gotimer K, et al (2009) The resting brain: unconstrained yet reliable. Cereb Cortex 19: 2209–2229. 19221144 10.1093/cercor/bhn256 PMC3896030

[5] Greicius M, Supekar K, Menon V (2008) Resting-state functional connectivity reflects structural connectivity in the default mode network. Cereb Cortex 19: 72–78. 18403396 10.1093/cercor/bhn059 PMC2605172

[6] Cools R, D’Esposito M (2011) Inverted-U-shaped dopamine actions on human working memory and cognitive control. Biol Psychiatry 69: e113–e125. 21531388 10.1016/j.biopsych.2011.03.028 PMC3111448

[7] Cools R, Gibbs S, Miyakawa A, Jagust W, D’Esposito M (2008) Working memory capacity predicts dopamine synthesis capacity in the human striatum. J Neurosci 28(5): 1208–1212. 18234898 10.1523/JNEUROSCI.4475-07.2008 PMC6671420

[8] Salthouse TA, Babcock RL (1991) Decomposing Adult Age Differences in Working Memory. Dev Psychol 27: 763–776.

[9] Daneman M, Carpenter P (1980) Individual differences in working memory and reading. JVLVB 19: 450–466.

[10] Patenaude BB, Smith SMS, Kennedy DND, Jenkinson MM (2011) A Bayesian model of shape and appearance for subcortical brain segmentation. NeuroImage 56: 16–16. 10.1016/j.neuroimage.2011.02.046 PMC3417233 21352927

[11] Jenkins GM, Watts DG (1968) Spectral Analysis and Its Applications. San Francisco: Holden-Day. 535 p.

[12] Lancaster JL, Woldorff MG, Parsons LM, Liotti M, Freitas CS, et al (2000) Automated Talairach atlas labels for functional brain mapping. Brain Mapp 10: 120–131. 10.1002/1097-0193(200007)10:3<120::AID-HBM30>3.0.CO;2-8 PMC6871915 10912591

[13] Maldjian JA, Laurienti PJ, Kraft RA, Burdette JH (2003) An automated method for neuroanatomic and cytoarchitectonic atlas-based interrogation of fmri data sets. NeuroImage 19: 1233–1239 (WFU Pickatlas, version 2.4).. 12880848 10.1016/s1053-8119(03)00169-1

[14] Kimberg D, D’Esposito M (1997) Effects of bromocriptine on human subjects depend on working memory capacity. Neuroreport 8: 3581–3585. 9427330 10.1097/00001756-199711100-00032

[15] Alexander GE, DeLong MR, Strick PL (1986) Parallel organization of functionally segregated circuits linking basal ganglia and cortex. Annu Rev Neurosci 9: 357–381. 3085570 10.1146/annurev.ne.09.030186.002041

[16] Fox PT, Lancaster JL (2002) Opinion: mapping context and content: the BrainMap model. Nat Rev Neurosci 3 (4): 319–321. 10.1038/nrn789 11967563

[17] Fox PT, Laird AR (2005) BrainMap taxonomy of experimental design: description and evaluation. Hum Brain Mapp 25 (1): 185–198. 10.1002/hbm.20141 PMC6871758 15846810

[18] Laird AR, Lancaster JL (2005) BrainMap: the social evolution of a human brain mapping database. Neuroinformatics 3 (1): 65–78. 10.1385/ni:3:1:065 15897617

[19] Robinson JLJ, Laird ARA, Glahn DCD, Blangero JJ, Sanghera MKM, et al (2012) The functional connectivity of the human caudate: an application of meta-analytic connectivity modeling with behavioral filtering. NeuroImage 60 (1): 117–129. 10.1016/j.neuroimage.2011.12.010 PMC3288226 22197743

[20] Kelly C, de Zubicaray G, Di Martino A, Copland D, Reiss P, et al (2009) L-dopa modulates functional connectivity in striatal cognitive and motor networks: a double-blind placebo-controlled study. J Neurosci 29: 7364. 19494158 10.1523/JNEUROSCI.0810-09.2009 PMC2928147

[21] Dodds CM, Clark L, Dove A, Regenthal R, Baumann F, et al (2009) The dopamine D2 receptor antagonist sulpiride modulates striatal BOLD signal during the manipulation of information in working memory. Psychopharmacology 207: 35–45. 19672580 10.1007/s00213-009-1634-0 PMC2764850

[22] Nagano-Saito A, Leyton M, Monchi O, Goldberg YK, He Y, et al (2008) Dopamine Depletion Impairs Frontostriatal Functional Connectivity during a Set-Shifting Task. J Neurosci 28: 3697–3706. 18385328 10.1523/JNEUROSCI.3921-07.2008 PMC6671089

[23] Wallace DL, Vytlacil JJ, Nomura EM, Gibbs SEB, D’esposito M (2011) The Dopamine Agonist Bromocriptine Differentially Affects Fronto-Striatal Functional Connectivity During Working Memory. Front Hum Neurosci 5: 1–6. 21503140 10.3389/fnhum.2011.00032 PMC3071499

[24] Honey GD, Suckling J, Zelaya F, Long C, Routledge C, et al (2003) Dopaminergic drug effects on physiological connectivity in a human cortico-striato-thalamic system. Brain 126: 1767–1781. 12805106 10.1093/brain/awg184 PMC3838939

[25] Fields HL, Hjelmstad GO, Margolis EB, Nicola SM (2007) Ventral tegmental area neurons in learned appetitive behavior and positive reinforcement. Annu Rev Neurosci 30: 289–316. 17376009 10.1146/annurev.neuro.30.051606.094341

[26] Chang C, Glover GH (2009) Effects of model-based physiological noise correction on default mode network anti-correlations and correlations. NeuroImage 47: 1448–1459. 19446646 10.1016/j.neuroimage.2009.05.012 PMC2995588

[27] De Mei CC, Ramos MM, Iitaka CC, Borrelli EE (2009) Getting specialized: presynaptic and postsynaptic dopamine D2 receptors. Curr Opin Pharmacol 9: 6–6. 10.1016/j.coph.2008.12.002 PMC2710814 19138563

